# The structure of global cultural networks: Evidence from the diffusion of music videos

**DOI:** 10.1371/journal.pone.0294149

**Published:** 2023-11-13

**Authors:** Marco Dueñas, Antoine Mandel

**Affiliations:** 1 Centre d’Economie de la Sorbonne - Paris School of Economics- CNRS-Université Paris 1 Panthéon-Sorbonne, Paris, France; 2 AXES Research Unit, IMT School for Advanced Studies Lucca, Lucca, Italy; ’Enrico Fermi’ Research Center, ITALY

## Abstract

We apply the independent cascade network inference model to a large database of music videos to infer the structure of the global network of music diffusion. The derived network reveals an intricate topology–fully interconnected, exhibiting a modular structure, and characterized by asymmetric links. We explore the relationship between the identified bilateral cultural diffusion pathways and the geographical and cultural distances among countries, and key socioeconomic interactions such as international trade and migration. Additionally, we use a gravity model to ascertain the factors contributing to both the formation and the intensity of the estimated diffusion channels between countries. Our findings reveal that cultural, geographical, and historical factors serve as primary drivers of musical diffusion, downplaying the importance of economic factors. This study posits that these elements exert considerable force in shaping musical preferences across nations, making the emergence of a homogeneous global musical culture improbable. This exploration adds valuable insights to the discourse on the globalization of music and its potential cultural implications.

## Introduction

Culture has played a key role in the globalization process [1–3]. Its impact is well documented on international trade [4], foreign direct investments [5], international business [6], and migration [7]. The converse impact of globalization on culture is more ambiguous [8, 9]. Three major theses on the issue are present in the scientific literature and the political discourse: homogenization, polarization, and hybridization [8, 10]. In particular, concerns about homogenization, or more specifically americanization [11], have had substantial political consequences with the emergence of cultural protectionism in many countries [12–15]. More broadly, capturing the extent and dynamics of cultural influence can offer valuable insights for policy evaluation, and integrating precise cultural information into economic analysis has the potential to enhance both cultural and economic analysis [16]. Nevertheless, little quantitative empirical evidence exists on the impact of globalization on culture. Our aim in this paper is to fill part of this gap by inferring the global network of music diffusion and analyzing its structure and the determinants of its formation.

Culture arises from the interactions of basic social units through imitative processes, where individuals copy and adopt the practices and beliefs of others [17, 18]. Hence, social networks and social influence play a crucial role in cultural diffusion [19]. Conventional linear models can hardly capture diffusion processes on such complex networks [20]. Furthermore, the empirical analysis of cultural diffusion requires comprehensive data on the prevalence of various interests and tastes across different regions and periods [16, 21]. Such data has only become available thanks to the digitalization of large parts of human activity [17].

Digitalization has notably impacted the production, consumption, and diffusion of music. It has led to substantial reductions in production, distribution, and promotion costs of new products [22]. The availability of a massive music library on platforms such as YouTube and Spotify has made it easier for people to discover and listen to music from different regions and cultures [23]. Furthermore, the increasing number of media diffusion channels, including the internet and social media, have led to a massive increase in the range and the speed of music diffusion [19, 24], as emphasized by the emergence of the notion of “virality” to describe these diffusion processes. Diffusion has further been catalyzed by institutional changes whereby markets have been less restricted by geopolitical boundaries and institutional decision-making processes [25]. This is due to the shift to a more digital form of globalization, which changes who participates, how business is done across borders, and where the economic benefits lie [26].

Diffusion of culture is less institutionalized than diffusion in other areas such as politics or economics. Gabriel Tarde’s *Laws of Imitation* [17] provides an interesting theoretical approach to the diffusion of culture, thanks to his focus on individual interests [27, 28]. According to Tarde, imitation is the primary mechanism by which culture is transmitted across generations, claiming that society is composed of beings that are apt to imitate one another, and even without actual imitation, they share traits that are ancient copies of the same model [17]. Therefore, imitation makes social change as individuals adopt new behaviors, beliefs, and norms. However, social imitation is not a straightforward copy of others; it involves a complex interplay between reason and extra-logical influences, such as social, psychological, and cultural factors. Furthermore, imitation can be accompanied by emulation, e.g., the world polity theory highlights that institutions conforming to a dominant world culture, characterized by universalism, rationalism, equality, and progress, are more likely to be emulated globally [29].

Tarde identifies various factors that can facilitate or obstruct the diffusion of cultural tastes and preferences. Individuals are more likely to imitate those similar to them in terms of cultural characteristics or individuals who possess prestige, status, or social influence. Conversely, pride could obstruct imitation besides cultural proximity. Despite the humble presence of Tarde’s laws of imitation in diffusion research [25], they provide a practical framework for understanding how cultural tastes and preferences spread across different social groups and make it possible to emphasize the importance of individual-level interactions and the role of social networks in shaping cultural influence.

While existing models of opinion formation propose that strengthened diffusion processes could lead to cultural homogenization [30, 31], considerable evidence suggests a preservation of substantial cultural diversity. Despite being prevalent across human populations, musical behaviors exhibit rich diversity in structure, role, and cultural interpretation [32]. International pop charts are increasingly diverse, incorporating more foreign music [33], even as interest in national artists grows [34, 35]. Simultaneously, national charts are diversifying, and countries previously close in taste are growing more similar, yet progressively distinct from others [36].

An emerging facet in this landscape is the persistence of group boundaries amidst rapid diffusion, delineating the local from the global [37, 38]. This notion aligns with previous research on video-sharing platforms like YouTube, which found that despite the ubiquity of diverse cultural products, consumption of popular videos is predominantly governed by cultural values [39]. These observations collectively point to the complex social interactions in play [19], where global broadcasting converges with interpersonal spreading [40], contributing to a nuanced interplay of cultural preservation and diffusion.

To study the characteristics and determinants of this complex interaction structure, we model the diffusion of popular music videos on YouTube as information cascades. As the largest existing video platform on the internet and holding the second position in popularity both as a search engine and a social network–with 2.6 billion users in 2022–YouTube provides an exceptional source of digital traces of human activity [41].

We use the information on all videos featured in the top 100 rankings of 57 countries, as reported weekly by YouTube Charts, from May 2019 to May 2022. We track the popularity and geographic diffusion of all new videos, looking at when they rank in different countries over time. Hence, to build the information cascades, we determine when the video was released and then track when it appears in countries’ charts. We then apply the independent cascade network inference model to discern the structure of the global network of music diffusion. The independent cascade model permits us to infer the most likely network of cultural diffusion among countries based on observed patterns of music video popularity.

The subsequent analysis of the inferred network furnishes quantitative insights about interaction structure, cultural distance, and clustering among countries, as well as the centrality and role of individual countries in the diffusion process. We further examine how the inferred bilateral cultural diffusion channels correlate with geographical and cultural distances between countries, along with other significant interaction channels such as international trade and international migration.

Finally, we employ a gravity model to determine how countries-specific and bilateral-specific features are related to the inferred network structure. The gravity model is a powerful tool used to estimate the presence and the degree of interaction between different actors, typically based on their size and geographical distance [42]. In particular, we delve into both the extensive and intensive margins of cultural diffusion. Here, the extensive margin refers to the factors determining the network formation of countries involved in the interactions, while the intensive margin concerns the intensity of these interactions.

## Results

### Identifying music video diffusion

In our study, the diffusion of a new music video starts when it is streamed on YouTube. As individual preferences aggregate into collective outcomes, certain videos become popular in one or multiple countries. To examine this phenomenon, we analyze aggregated data at the country level, specifically focusing on music videos that have reached the top 100 charts in different countries. We consider new music videos from May 2019 to May 2022 in country YouTube Charts, we follow them until August 2022, identifying 57,439 videos.

We consider that the diffusion of music videos can be analyzed as information cascades at the country level. This occurs when individuals in a particular country rely on the actions or decisions of others in their country and in foreign countries when making their own decisions about watching/listening a music video. In this context, as more and more individuals in the country follow the behavior of others, it creates a cascade that can ultimately lead to widespread adoption or rejection of the music video within that country. Therefore, it is important to note that with the country aggregated rankings data we possess, we cannot directly observe granular diffusion paths dictating which viewers directly influence others to watch/listen specific videos. Instead, we shall aim to infer potential pathways for such aggregated diffusion, based on observable adoption patterns. This caveat acknowledges the inherent limitations of our data and reinforces our aim to discern potential, rather than definitive, patterns of influence and adoption.

We find that music video diffusion is strikingly heterogeneous concerning the number of views, the cascade size–the number of countries where a video reaches popularity–and the cascade duration–the time it takes to achieve its maximum geographic scope. While most countries exhibit high receptivity to international music videos, in certain countries like Turkey, Japan, Romania, Egypt, Israel, India, and Korea, merely around 25% of viewed videos are international (see Fig S1, in S1 Appendix).

Several factors may account for this observed pattern. Unique languages certainly play a role in shaping viewing preferences, given that the language barrier can restrict the appeal of foreign content. Moreover, countries like Japan, Korea, and India are recognized for their strong domestic music industries, which create significant local content. The prominence of such homegrown music can contribute to a cultural environment where international videos might be less predominant.

Cultural protectionism can also shape this trend. Less receptive countries may have policies that favor the promotion of local over foreign content, whether as part of a formal strategy to preserve national culture or due to pressure from local industries to maintain their market share [12, 13, 15]. These actions might limit the exposure and accessibility of international music videos, thereby influencing viewing habits.

**Fig 1:**
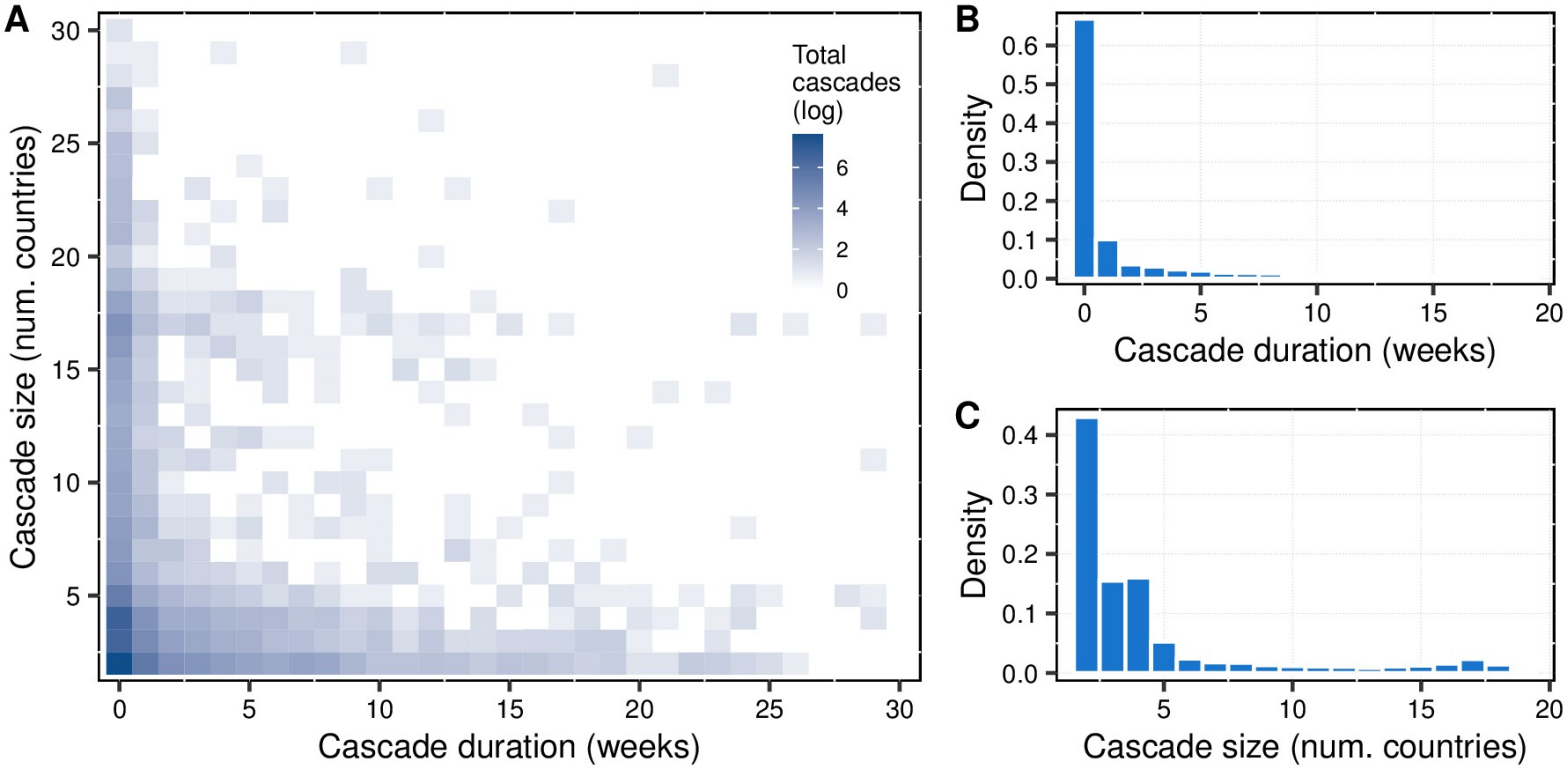
Diffusion cascades and cascade duration and size distributions. Panel A: Heat map of all cascades in the sample, in the x-axis the cascade duration (the length of time that it takes to spread to its maximum geographic scope), and in the y-axis the cascade size (geographic scope). Panel B: The density distribution of the cascade duration. Panel C: The density distribution of the cascade size.

We recognize three different domains of diffusion of popular videos: i) within-country diffusion and non-internationalization, these are the videos that reach only one country; ii) speedy international diffusion, these are the videos that reach the maximal popularity in just one week, i.e., cascades with a single synchronized adoption time, quite similar to a pure broadcasting event; and, iii) international diffusion with more than one subsequent adopter, these are cascades of videos that diffuse gradually through different countries. Fig 1A portrays the number of cascades in terms of size and duration for the last two domains, which account for 6,977 videos–for obvious reasons, we excluded the first domain since it does not provide any insight into international diffusion (data is available in S1 Data).

Considering the worldwide broadcasting nature of YouTube, many of the international cascades reach their maximum popularity within a few weeks (see Fig 1B). A vast majority of cascades belong to the second domain, about 64%, implying that the root nodes and the immediate adopters are indistinguishable. In the third domain, 16% of international cascades diffuse within two and four weeks and 10% within five and ten weeks. Also, it is quite unlikely that a single song will become worldwide popular (see Fig 1C). In contrast, international popularization frequently happens in a small number of countries. About 40% of the songs are popularized in two countries, 30% between three and four countries, and 10% between five and eight countries. Less than 3% of the songs become popular in more than 30 countries, and less than 0.2% reach popularity in the total sample of countries.

### The cultural diffusion network

We use international cascades in the third domain to infer the diffusion network across countries, with 2,487 videos. We use the information propagation model proposed by Gomez-Rodriguez et al. (2014) [43]. This inference model assumes that adoptions occur independently along the network’s edges and that the likelihood of adoption depends on the influencer node’s identity, the adopting node’s identity, and the time of adoption. Based on the observed adoption times, the model aims to infer the network’s connectivity and the likelihood of adoptions across its edges.

In this context, it is crucial to clarify our interpretation of “influence” between countries, given the nature of our model and data. Music videos in our sample are not assigned a specific nationality–a task that is not relevant for our study, and yet it can be complex considering the increasing cross-national collaborations and the prevalence of international artists for whom assigning a nationality is problematic. Consequently, our reference to “influence” should not be misconstrued as a country imposing its cultural artifacts or specific artists onto others. Instead, we argue that the diffusion of music videos on the internet reflects a form of cultural diffusion, more specifically, the propagation of musical preferences or tastes between different countries. These preferences, unlike artists, do not necessarily need a nationality. As an example, while direct adoption of Latin American music might be less probable in most European countries, Spain and Portugal often act as initial adopters due to cultural and linguistic ties. These countries, which potentially have better diffusion channels towards other European nations, can catalyze the introduction and acceptance of Latin American music throughout Europe. Therefore, the inferred network is more about tracing the pathways of shared tastes and preferences rather than tracking the expansion of specific national cultural products.

**Fig 2:**
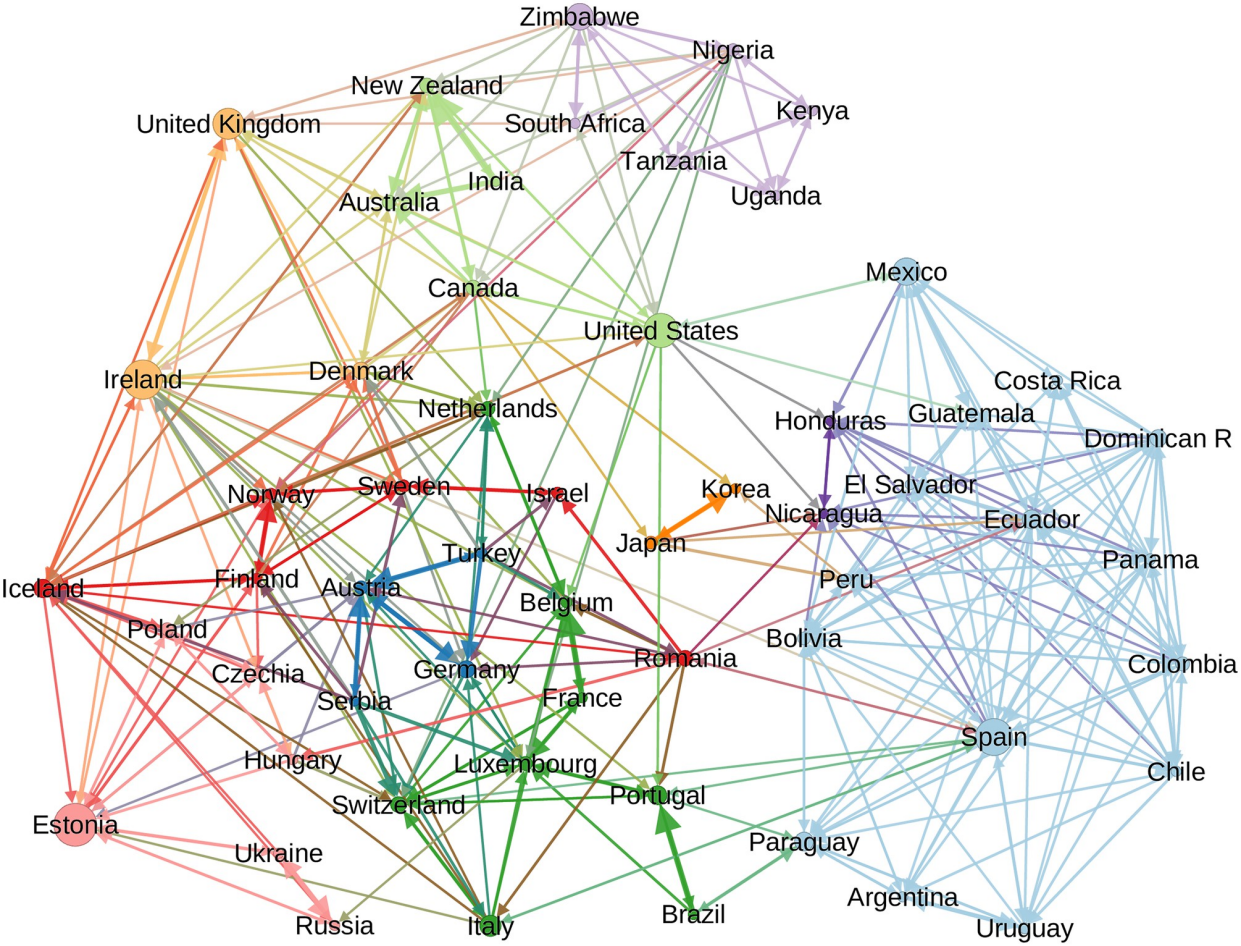
International cultural diffusion network. Nodes represent countries and links the most likely cultural diffusion channels, taking as a proxy the estimated transmission rates. Node’s size indicates betweenness. Detected communities are depicted in different colors.

Fig 2 shows the inferred network, which provides the paths of the diffusion routes and, hence, a complete view of the worldwide bilateral influence transfers. The estimated network exhibits a complex topological structure with no global influencer or hierarchy (in S1 Appendix, we present an alternative estimation of the network, underlying the robustness of our findings: see Fig S4 in S1 Appendix). The overarching structure is characterized by its fully connected nature, incorporating a modular structure and the presence of asymmetric links. The estimated network is reported in S2 Data.

The link weights’ distribution is skewed to the right and takes very low values (see Fig S2 in S1 Appendix); with a significant proportion of them approaching zero for many country pairs, which implies a null influence. Consequently, the network is sparse: only 12% of the possible connections exist. The estimated link weights are very asymmetric: the network reciprocity index is barely 0.37 [44]. These values are relatively low compared to, for example, the international trade network [45], which has a density of 98% and a reciprocity index of 0.92 (we built the international trade network using the same sample of countries in 2019).

The estimated network is relatively highly modular. We use the Infomap Algorithm to detect communities [46], the modularity is 0.59, and there are ten detected communities, which are also depicted with different colors in Fig 2 (for the list of countries in communities, see Table 1). The modular structure gathers communities of countries that, in most cases, are geographically close and share a common language. The largest community is made up of Latin American countries and Spain. At the top of the graph, there are English-speaking countries, composed of a community of African countries, another community with some former British colonies and the United States, and the United Kingdom, Ireland, and Denmark forming a small additional community. On the left part of the center of the graph, the countries of continental Europe are in different communities. There is a community with countries from Northern Europe, close to the English-speaking countries in the graph (that includes Romania and Israel), and two other communities whose languages derive from the Romance and German-speaking countries, plus Turkey and Serbia. On the bottom left side of the graph, there are Eastern European countries whose languages derive from the family of Slavic languages. And, finally, two small communities, Japan-Korea and Honduras and Nicaragua. To identify some relevant countries in terms of centrality and their quality of influencer or influenced, Table 1 also provides information about the countries reaching the highest betweenness centrality per community, as well as the countries that have the highest out- and in-strength, differentiating between intra- and inter-community interaction.

**Table 1:** Detected communities and highlighted countries based on selected network metrics.

Community members	Size	Betweenness*	Intra-community out-strength*	Inter-community out-strength*	Intra-community in-strength*	Inter-community in-strength*
Argentina; Bolivia; Chile; Colombia; Costa Rica; Dominican Rep.; Ecuador; Spain; Guatemala; Mexico; Panama; Peru; Paraguay; El Salvador; Uruguay	15	Spain (688)	Spain (0.41)	Spain (0.06)	Uruguay (0.29)	Paraguay (0.07)
Austria; Germany; Serbia; Turkey	4	Germany (177)	Turkey (0.28)	Serbia (0.43)	Austria (0.42)	Austria (0.12)
Australia; Canada; India; New Zealand; United States	5	United States (618)	India (0.45)	United States (0.12)	New Zealand (0.42)	Australia (0.07)
Belgium; Brazil; Switzerland; France; Italy; Luxembourg; Netherlands; Portugal	8	Italy (279)	France (0.36)	Italy (0.07)	Belgium (0.34)	Switzerland (0.25)
Czech Rep.; Estonia; Hungary; Poland; Russian Federation; Ukraine	6	Estonia (840)	Ukraine (0.22)	Poland (0.1)	Estonia (0.18)	Estonia (0.11)
Denmark; United Kingdom; Ireland	3	Ireland (747)	Ireland (0.14)	Ireland (0.21)	United Kingdom (0.14)	Denmark (0.14)
Finland; Israel; Iceland; Norway; Romania; Sweden	6	Iceland (207)	Finland (0.23)	Romania (0.23)	Norway (0.25)	Iceland (0.2)
Honduras; Nicaragua	2	Honduras (0)	Honduras (0.03)	Honduras (0)	Nicaragua (0.03)	Honduras (0.15)
Japan; Rep. of Korea	2	Japan (65)	Japan (0.15)	Japan (0.09)	Rep. of Korea (0.15)	Rep. of Korea (0.01)
Kenya; Nigeria; United Rep. of Tanzania; Uganda; South Africa; Zimbabwe	6	Zimbabwe (423)	United Rep. of Tanzania (0.18)	Zimbabwe (0.05)	Kenya (0.16)	South Africa (0.02)

*Notes:* Communities detected using the Infomap Algorithm [46]. Columns marked with * indicate the countries within each community that achieve the maximum values for the corresponding network statistic, values reported in parenthesis.

The binary network diameter–the shortest distance between the most distant nodes–is seven edges, and the weighted network diameter–the path with the lowest total weight–is 0.26. This implies that while many paths can connect the most distant nodes, the path with the lowest total weight is 0.26. In other words, international diffusion between the two most distant nodes would be in the most favorable scenario if a new popular video goes through 7 edges. However, as expected from the link weight distribution, this could happen with a very low probability since each edge is associated with a low transmission rate.

The potential for one country to exert substantial influence over another is rather constrained. This suggests that, despite the fully connected nature of the network, numerous factors–such as sparse levels of interaction, asymmetrical links between countries, and nodes segregated into distinct communities–substantially impede the homogenization of musical tastes. In essence, these elements obstruct a uniform diffusion of music preferences across diverse countries.

Besides, we examine the similarity of country music charts of videos in the second domain (speedy international diffusion). Although this approach does not allow us to discern the potential influence of one country over another, it does encompass all possible paths of influence. Interestingly, we find that the similarity network closely resembles the inferred cascade network, particularly in terms of the neighborhood structures. This congruence strengthens our confidence in the inferred network, suggesting that, despite the absence of granular influence data, the inferred network is a meaningful representation of potential influence pathways. This analysis is presented in S1 Appendix.

### Correlation patterns

**Fig 3:**
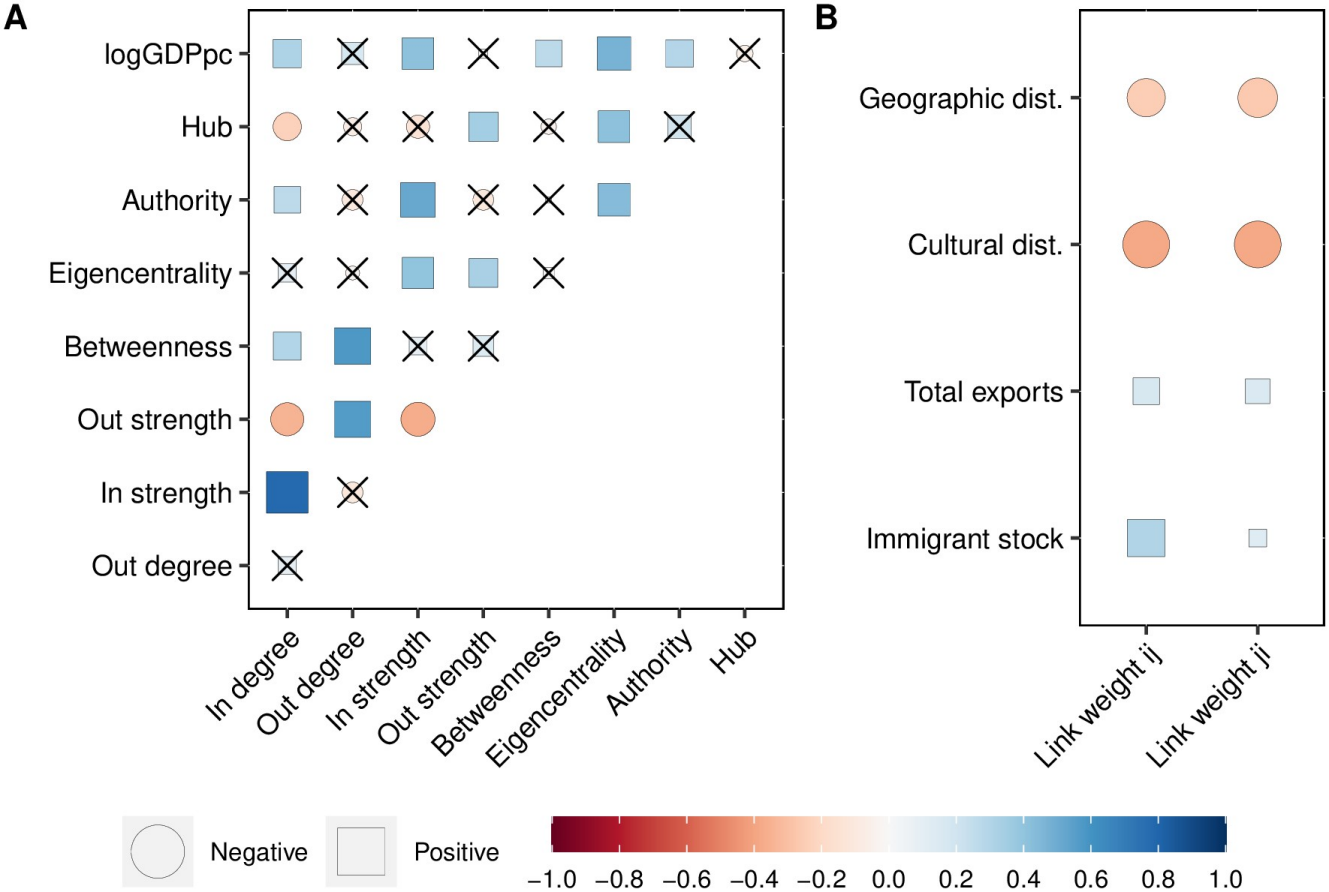
Correlation plot. Panel A: Node network statistics correlations, including centrality measures and GDPpc. Panel B: Link level correlations, considering bilateral international trade and migration stocks, cultural and geographical distance. Cross-out signs imply not statistically significant correlations at a *p*-value of 0.01.

Fig 3A shows the correlations between network statistics and the nodes’ Gross Domestic Product per capita (GDPpc). The correlations between degree and strength within the same in- or out-orientation are positive, as expected, meaning that the country’s influence or level of being influenced is related to the strength of interactions with its outgoing and incoming neighbors, respectively. However, the negative correlations between the in- and out-statistics indicate that the diffusion network is highly directional. Likewise, the low reciprocity implies that in a bilateral cultural exchange, one of the countries is typically more influential. In Tarde’s view, this could mean that some nodes are perceived as more prestigious by their neighbors, and this could activate imitation, whether by emulation or social learning.

There are positive correlations among betweenness centrality and both in- and out-degree and strength, although not significant for node strength. This implies that the nodes through which most of the information is expected to flow are the most connected but not necessarily the most influential or influenced. For other centrality measures such as eigenvector, authority, and hub centralities [47], we find that they correlate differently with the in and out node statistics, pointing out that nodes’ centrality functions within the network are very heterogeneous. Eigenvector centrality, which measures the influence of a node, taking into account the quality of its connections, is significantly correlated with the strength sequences. We also consider Authority and Hub centralities. Authority measures a node’s importance based on hubs pointing to it, while Hub assesses how well a node directs others to relevant authorities. We found that authority centrality correlates positively with in-strength; this is that highly influenced countries tend to have higher authority centrality, which means that these countries are reference sources of authoritative information. In addition, hub centrality correlates with out-strength; therefore, the most influential countries tend to have higher hub centrality, which means that these countries serve as reference points or sources of authoritative information.

Further, representative Western culture countries, such as the United States, United Kingdom, France, or Germany, are not the most connected in the network. Although there is a positive correlation between income level (GDPpc) and the in-degree and strength, suggesting that the higher the income, the stronger the external influence, the correlations of income with the out-degree and strength are not significant. This differs from international trade, where rich economies have more trading partners and higher trade flows [45].

Notwithstanding, income positively correlates with other centrality measures, such as betweenness, eigenvalue centrality, and authority. Therefore, even when high-income countries are not necessarily influencers, they play an important role in worldwide diffusion. For example, countries like Uganda, Ukraine, and Argentina are far apart in the network (see Fig 2); however, several high-income countries within the intermediate neighbors could facilitate the cultural diffusion between these countries.

Further, we examine the correlations of the link weights with bilateral exports and bilateral stocks of migrants in two senses: the direct and the reverse relationship (see Fig 3B). In the direct, we ask for the correlation between the transmission rate of country *i* on country *j* with the total amount of exports from *i* to *j* and the stock of immigrants of *i* in *j*. While in reverse, we correlate the transmission rate of *j* on *i* with total exports from *i* to *j* and the stock of immigrants of *i* in *j*. In addition, we use a bilateral cultural distance variable that measures the bilateral differences in attitudes toward authority, trust, individuality, and importance of work and family [48], and the geographical distance, which is the great circle distance between the geographic center of countries.

Concerning the immigration stocks of origin *i* in destination *j*, we find significant direct and reverse correlations of 0.30 and 0.13, respectively. This means that the influence strength of *i* in *j*, could be determined by the stock of immigrants from country *i* in country *j*, and at the same time, this stock of immigrants might cause the influence of country *j* on country *i*. In other words, the evidence suggests that there might be a feedback loop between immigration and cultural diffusion, where the presence of immigrants from a specific country can increase the influence of their home country on the destination country and the influence of the destination on the home country.

Similarly, for the total bilateral exports from *i* to *j*, we find significant direct and reverse correlations of 0.16 and 0.15. These results indicate that bilateral trade might strengthen mutual cultural diffusion. The study of the relationship between trade and culture has a long tradition in the international economics literature [4]. Culture is seen as a trade barrier when exporters and importers are distant in terms of language or colonial linkages. Although the purpose of this paper is far from determining causality between cultural differences and trade, it is interesting to find a significant and positive correlation with trade. Our estimations can represent an alternative assessment of cultural differences to understand international non-tariff trade barriers, which are typically difficult to observe.

A negative correlation suggests that the greater the cultural distance between two countries, the less influence they exert on each other’s music preferences. While this measure of cultural distance is only available for a select group of countries [48], the observed correlation implies that similarities in attitudes toward authority, trust, independence, government, family, and work positively relate to the rate of music preference transmission. Additionally, geographical proximity appears to facilitate diffusion, as indicated by the negative correlation with geographical distance.

### Determinants of bilateral diffusion

We estimate a gravity model to test how the intensity of cultural diffusion is related to economic and demographic characteristics of the countries, such as language similarity, geographical proximity, and historical ties. We model the emergence of cultural diffusion linkages with a Logit model and their intensity using a Zero-Inflated Negative Binomial (ZINB) model. We aim to identify the relative importance of different factors in shaping the cultural relationship between country pairs.

The results of our analysis suggest that both the Logit model and the ZINB model provide good fits for the data (see Table 2). We find that nodes’ income plays a mild role in the network’s formation and has a greater impact in determining the interaction’s intensity. Regarding link formation, the marginal effect of income (at average levels) of both the source and the destination on the probability of link formation is around 1.4% (see column 2 in table). Instead, concerning the intensity of the interaction, we found that, for a given dyad, the incidence rate ratio (IRR) of the source’s income is 0.821, and of the destination’s income is 1.270 (see column 5 in table). This implies that countries’ income significantly affects the intensity of the interaction once a link is formed, with the destination’s income having a larger effect than the income of the source.

**Table 2:** Gravity model estimations.

	Extensive Margin		Intensive Margin (ZINB)		
	(1)	(2)	(3)	(4)	(5)
	Logit	Marginal Effect	2nd Stage	1st Stage	IRR (2nd stage)
ln *GDPpc*_*o*_	0.268***	0.014***	-0.198**	-0.277***	0.821**
	(0.061)	(0.003)	(0.078)	(0.062)	(0.064)
ln *GDPpc*_*d*_	0.257***	0.014***	0.239***	-0.253***	1.270***
	(0.063)	(0.003)	(0.060)	(0.063)	(0.076)
ln Geo. Distance	-0.789***	-0.043***	-0.101*	0.789***	0.904*
	(0.075)	(0.004)	(0.058)	(0.075)	(0.052)
Contiguitity_*od*_	0.490*	0.026*	0.468***	-0.492*	1.597***
	(0.280)	(0.015)	(0.133)	(0.282)	(0.212)
Common Language_*od*_	2.486***	0.134***	0.198	-2.497***	1.219
	(0.169)	(0.010)	(0.124)	(0.170)	(0.152)
Colony_*od*_	1.005***	0.054***	0.554***	-0.994***	1.740***
	(0.273)	(0.015)	(0.172)	(0.274)	(0.300)
Common Colonizer_*od*_	0.866**	0.047**	0.284	-0.868**	1.328
	(0.375)	(0.020)	(0.178)	(0.379)	(0.237)
Constant	-1.495	3.324***	1.515		
	(1.268)	(0.722)	(1.279)		
Observations	3,080	3,080	3,080	3,080	

*Notes:* The dependent variable of the extensive margin is whether *w*_*od*_ > 0. The dependent variable of the intensive margin is *w*_*od*_. Robust standard errors are in parenthesis. Significance level: *** p<0.01, ** p<0.05, * p<0.10. The log of the estimated dispersion parameter by the ZINB model is ln *α* = -0.553***(0.098).

Geographical distance harms both the formation and the intensity of links. Therefore, distant countries are less likely to influence each other. In contrast, the variables related to sharing a common border, common official language, common colonizer, and colonial relationship have a positive and significant effect. Having a common border contributes to the link formation in a 2.6%, and the IRR is 1.597, i.e., the interaction is 60% stronger than that of two countries that are not contiguous.

Common language is the variable with the greatest impact on link formation. Two countries with the same language are 13.4% more likely to influence each other, and this interaction has an IRR of 1.219. A historical colony relationship contributes to the link formation in 5.4%, with an IRR of 1.740. Sharing a common colonizer contributes link formation in 4.7%, with an IRR of 1.328. These results imply that there may be lingering cultural and economic ties between countries with a shared history, which can affect their current interactions.

## Discussion

With the internet and social networks, the spread of different cultural forms worldwide is a topic of great interest. In this paper, we use cascades of information from popular music videos and a network inference methodology to quantitatively assess the cultural diffusion between countries. The inferred network is sparse, has low reciprocity levels, and is characterized by a modular structure. In this network, there is no outstanding influencers, which indicates that the influencing strength of musical tastes between countries is limited. However, the presence of modules indicates that the musical influence occurs more likely within these groups than outside them. Additionally, we investigate possible factors determining the link interactions. We find that macroeconomic variables, such as income, are comparatively less relevant than geographic, cultural, and historical factors to explain the link formation and intensity of international diffusion.

The ideas presented by Tarde [17] provide a sound theoretical explanation for our findings in at least two important regards. First, the low reciprocity between dyads shows that, typically, there is an influencer and an influenced country, meaning that countries have different profiles at the bilateral level: one country has more “prestige”, so the other country is prone to learn and imitate its actions. Second, the significant presence of low influence and the modular structure reveals that cultural factors limit learning and imitation. This means that culture itself generates barriers to coordinate the diffusion.

An interesting finding is the relatively minor importance of countries’ income against other variables linked to cultural differences. In other channels of interaction between countries, for example, international trade, developed countries play a central role [45]. Historically, international trade has been considered the economic variable most closely linked to globalization. If we would adopt Tarde’s ideas for the international trade network, then income and development would be good indicators of prestige, and countries would imitate strategies to achieve development. The international trade network has something similar to a core-periphery structure, where the countries at the core have higher GDP, and those at the periphery are less developed [45]. However, this pattern is not emulated in the diffusion network we estimated here.

Nonetheless, one may wonder about the role of the richer countries in the cultural influence worldwide. Although the fear of the globalization of the Western culture continues to be a matter of concern [11], our evidence points in the other direction: high-income countries are comparatively more influenced. Beyond the positive correlation between node’s in-strength and income, high-income countries have a greater variety of videos in their charts–consequently, are more culturally receptive–and have higher centrality statistics, such as betweenness, eigenvector, and authority. All this indicates that more than promoters of their own culture, the greater exposure of these countries makes the network much more cohesive since they are also intermediary neighbors that connect distant countries with much more diverse preferences for music videos.

The empirical evidence we presented takes place in a context where digitalization has notably impacted music production, consumption, and diffusion. Although we covered the weekly top 100 songs in countries’ charts according to YouTube, other relevant streaming channels such as Spotify or iTunes exist. The availability of all these massive music libraries with the combinations of social media has increased the speed of music diffusion [19, 24]. It is worth mentioning that music streaming platforms not only grappled with the deeply implanted music industry of powerful multinationals but also with the sovereign institutional and geopolitical decisions of nations, to which the copyright legislation and the negotiation with producers and artists played a key role [49–51].

As a final remark, we acknowledge that cultural differences can vary from subtle nuances to obvious characteristics. Nevertheless, estimating the magnitude of cultural influence is a complex task, making it essential to implement new methodologies and use innovative information sources. Certain cultural attributes can provide additional information to understand the diffusion process and international influence. For instance, languages that are more geographically proximate, more historically related, and/or spoken by more-similar cultures have more aligned word meanings [52], showing that language can be described more extensively. The similarity in musical preferences, rather than relating to songs by specific artists, can be generalized to sensory characteristics and music universals, which are related to features such as pitch, rhythm, and performance style. Cross-cultural structural regularities of human music may relate to roles in facilitating group coordination and cohesion [53], and variations in acoustic features can highlight key differences in the habits of cultures [54]. Therefore, the heterogeneity in how music is perceived can also be a limiting factor to international influence, which relates to the traditional music experiences in countries.

Our estimated international network of cultural diffusion provides a perspective on assessing international cultural influence, yet it represents only one potential approach among many to understand these dynamics. The nature of the information diffused can greatly influence the characteristics of the diffusion process. For instance, distinct forms of content, such as images, videos, news stories, and petitions, have remarkably different diffusion patterns on platforms like Twitter [40]. Moreover, analyses of Google Trends data concerning the top 10 trending topics reveal that cultural interests and consumer tastes are predominantly country-specific and not broadly shared across regions [25]. Therefore, it is important to underscore that our results represent a snapshot of a complex and dynamic system. In the long-term perspective, the influence and diffusion of culture can be significantly affected by other forms of international interaction [1]. Notably, global phenomena such as international trade and migration have been shown to generate cultural exchanges and modify culture over time. Hence, a comprehensive understanding of cultural dynamics should integrate these factors into its analysis. The exploration of these multifaceted relationships will be a fruitful direction for future research, helping to build a more nuanced understanding of cultural evolution and influence in an increasingly interconnected world.

## Methods

### Data

Our study compiles data for all videos featured in the top 100 rankings, as reported weekly by YouTube Music Charts & Insights (Top songs), from May 2019 to May 2022, which are publicly available online. To our knowledge, the collection and analysis of this data comply with YouTube’s terms and conditions for data usage. Our database comprises information from 57 countries across the globe (see S1 Appendix for the list of countries and the cascades used in the analysis). We have weekly time-series data for each video that encompasses its ranking position and the number of views in each country where the video made it to the top charts. We use the YouTube API (application programming interface) to determine the video’s publication date and select the new music videos only. Using this information, we determine the activation time, defined as the moment when a video reaches its peak number of views for each country-video time series. This enables us to build the complete set of cascades. We additionally use GDPpc data from the World Bank [55], bilateral trade data from CEPII [56] and international migrant stock data from UNDESA [57].

#### Network inference

We implement an independent cascade model to infer the network of cultural diffusion [43]. Our model relies on the latent link variables as the independent variables, which are a set of link dummies, to capture the unobserved cultural interactions that govern the bilateral diffusion between countries. Each music video *c* has a cascade of adoptions $t^{c}=\left(t_{1}^{c},\ldots ,t_{N}^{c}\right)$, which is an N-dimensional vector of observed activation times, representing the time at which each country adopted the music video. For each country *i*, $t_{i}^{c}$ is an element of $[t_{0}^{c},t_{0}^{c}+T]\cup \infty$. This means that $t_{i}^{c}$ equals the time at which country *i* adopted the music video *c* if the adoption happened during a time interval of length *T* starting with the first adoption at time $t_{0}^{c}$. If country *i* did not adopt the video during this interval, then $t_{i}^{c}$ is infinite. Then, all video songs can be represented by a set **C** of cascades, one cascade for each music video, and denoted as **C** ≔ {**t**^1^, …, **t**^|*C*|^}.

Our goal is to use **C** to deduce an diffusion network represented by a pair (*G*, *A*), where *G* = (*V*, *E*) is a graph consisting of nodes *V* and edges *E* that represent potential cultural diffusion paths. The matrix *A* = [*α*_*ji*_] contains transmission rates, with *α*_*ji*_ ≥ 0 representing the likelihood that a video will spread from node *j* to node *i* if (*j*, *i*) ∈ *E* (and *α*_*ji*_ = 0 if (*j*, *i*) ∉ *E*).

The independent cascade model assumes that each cascade is an independent diffusion process and aims to infer the maximum likelihood network based on this assumption. The probability of diffusion from node *j* to node *i* is parameterized by the transmission rate *α*_*ij*_ and denoted as *f*(*t*_*i*_|*t*_*j*_; *α*_*ji*_). This probability is used to infer the likelihood of a set of cascades given a network *A* = [*α*_*ji*_]. The functional form of *f* conveys the structural assumptions about the diffusion process. Here, the function *f*(⋅) chosen is based on a Poisson process, meaning that the probability rate at which one country influences another remains constant over time once a video becomes popular in a country. This leads to an exponential model for the conditional diffusion density over time, more precisely *f*(*t*_*i*_|*t*_*j*_; *α*_*ji*_) = *α*_*ji*_*e*^−*α*_*ji*_(*t*_*i*_ − *t*_*j*_)^. This Poisson assumption is a simple and natural benchmark when there is no specific information available about the dynamic aspects of the diffusion strategies in the fine-grained structure [58]. In S1 Appendix, we explore an alternative functional assumption related to the diffusion process. Instead of the exponential form, we considered a power-law, defined as $f\left(t_{i}|t_{j};\alpha_{ji}\right)=\alpha_{ji}{\left(t_{i}-t_{j}\right)}^{1-\alpha_{ji}}$. This alternative assumption suggests that while transmission occurs very rapidly, there’s still a significant possibility of observing extended transmission times. The resulting estimated network based on this assumption is illustrated in Fig S4 in S1 Appendix.

Let *f*(**t**^*c*^; *A*) be the likelihood of a cascade *c*, then we aim to resolve the following maximum likelihood optimization problem:

$$\begin{array}{c} \begin{array}{cc} & \underset{A}{\text{min}}-\sum_{c\in C}\text{log}\;f\left(t^{c};A\right)\;;\\ & \text{subject}\;\text{to}\;\alpha_{ji}\geq 0,\text{for}\;i,j=1,\ldots ,N;i\neq j\;. \end{array} \end{array}$$ (1)

For details see S1 Appendix.

#### Econometric model

We estimate a gravity model to analyze the cultural influence among countries. The gravity model is a widespread tool for studying the flows of goods, people, and information across different regions (see, e.g., [59]). A common issue with such models is the presence of a large number of zeros in the dependent variable, which can be attributed to factors such as spatial barriers or socioeconomic constraints. In order to account for this excess of zeros, we adopt a Zero Inflated Model (ZIM) framework, which allows us to model at the same time the count of non-zero observations and the excess of zeros. The ZIM provides a more accurate and robust estimation of the parameters, as it considers the probability of an event to be zero or non-zero.

More precisely, we investigate the link formation and link weights using the following specification:

$$\begin{array}{c} y_{od}=F\left(\beta_{0}+\beta_{1}\;\text{ln}\;X_{o}+\beta_{2}\;\text{ln}\;X_{d}+\beta_{3}\;\text{ln}\;D_{od}+\theta \cdot Z_{od}\right); \end{array}$$ (2)

where, *y*_*od*_ is either the estimated link weight intensity *w*_*od*_, of origin country *o* on destination country *d*, or the probability this link exists Pr[*w*_*od*_ > 0|⋅], i.e., the intensive and the extensive margins correspondingly. *X* is the GDPpc; *D* is the geographical distance; and *Z* is a set of bilateral dummies: contiguity, common language, common colonizer, and colony relationship. The function *F*(⋅) references the estimation method according to the left-hand variable: Zero-Inflated Negative Binomial (ZINB) model for the intensive margin and Logit model for the extensive margin. For more details see S1 Appendix–estimations suggest that the Zero Inflated Negative Binomial model performs better than the Zero Inflated Poisson model.

## Supporting information

S1 Appendix Supplementary descriptive statistics, methods, and results. (PDF)

S1 Data Network inference cascades dataset. (CSV)

S2 Data Inferred network dataset. (CSV)

## References

[1] Appadurai A. Modernity at large: Cultural dimensions of globalization. vol. 1. University of Minnesota Press; 1996.

[2] Gilardi F. Transnational diffusion: Norms, ideas, and policies. In: Carlsnaes W, Risse T, Simmons BA. Handbook of international relations. 2012;2:453–477.

[3] Fukuyama F. Political order and political decay: From the industrial revolution to the globalization of democracy. Macmillan; 2014.

[4] Head K, Mayer T. What separates us? Sources of resistance to globalization. Canadian Journal of Economics/Revue Canadienne d’Économique. 2013;46(4):1196–1231. doi: 10.1111/caje.12055

[5] Erel I, Jang Y, Weisbach MS. Cross-border mergers and acquisitions. National Bureau of Economic Research; 2022.

[6] Hofstede G. Culture’s consequences: International differences in work-related values. vol. 5. SAGE; 1984.

[7] Lanati M., Venturini A. Cultural change and the migration choice. Review of World Economics. 2021;157(4):799–852. doi: 10.1007/s10290-021-00418-1

[8] Holton R. Globalization’s cultural consequences. The Annals of the American Academy of Political and Social Science. 2000;570(1):140–152. doi: 10.1177/0002716200570001011

[9] Brady D, Beckfield J, Zhao W. The consequences of economic globalization for affluent democracies. Annual Review of Sociology. 2007;33:313–334. doi: 10.1146/annurev.soc.33.040406.131636

[10] Holton RJ. Globalization’s cultural consequences revisited. In: Robertson R, Buhari-Gulmez D. Global culture: Consciousness and connectivity. Routledge; 2017;p.55–74.

[11] Sznaider N, Winter R. Global America?: The cultural consequences of globalization. Liverpool University Press; 2004.

[12] Baughn CC, Buchanan MA. Cultural protectionism. Business Horizons. 2001;44(6):5–16. doi: 10.1016/S0007-6813(01)80068-9

[13] Kish KA. Protectionism to promote culture: South Korea and Japan, a case study. U Pa J Int’l Econ L. 2001;22:153.

[14] Brooks E. Cultural Imperialism vs. Cultural Protectionism: Hollywood’s Response to UNESCO Efforts to Promote Cultural Diversity. Journal of International Business and Law. 2006;5(1):5.

[15] Burri M. Cultural protectionism 2.0: Updating cultural policy tools for the digital age. In: Pager SA, Candeub A. Transnational culture in the internet age. Edward Elgar Publishing; 2012.

[16] Adkisson RV. Quantifying culture: problems and promises. Journal of Economic Issues. 2014;48(1):89–108. doi: 10.2753/JEI0021-3624480104

[17] Tarde G. The laws of imitation. H. Holt; 1903.

[18] Latour B, Jensen P, Venturini T, Grauwin S, Boullier D. ‘The whole is always smaller than its parts’–a digital test of Gabriel Tardes’ monads. The British Journal of Sociology. 2012;63(4):590–615. doi: 10.1111/j.1468-4446.2012.01428.x 23240834

[19] Susarla A, Oh JH, Tan Y. Social networks and the diffusion of user-generated content: Evidence from YouTube. Information Systems Research. 2012;23(1):23–41. doi: 10.1287/isre.1100.0339

[20] Boccaletti S, Latora V, Moreno Y, Chavez M, Hwang DU. Complex networks: Structure and dynamics. Physics Reports. 2006;424(4-5):175–308. doi: 10.1016/j.physrep.2005.10.009

[21] Bail CA. The cultural environment: Measuring culture with big data. Theory and Society. 2014;43:465–482. doi: 10.1007/s11186-014-9216-5

[22] Waldfogel J. How digitization has created a golden age of music, movies, books, and television. Journal of Economic Perspectives. 2017;31(3):195–214. doi: 10.1257/jep.31.3.195

[23] Hracs BJ, Webster J. From selling songs to engineering experiences: exploring the competitive strategies of music streaming platforms. Journal of Cultural Economy. 2021;14(2):240–257. doi: 10.1080/17530350.2020.1819374

[24] Gomez-Herrera E, Martens B, Waldfogel J. What’s Going On? Digitization and Global Music Trade Patterns since 2006. Digitization and Global Music Trade Patterns Since. 2006;.

[25] Bail CA, Brown TW, Wimmer A. Prestige, proximity, and prejudice: how Google search terms diffuse across the world. American Journal of Sociology. 2019;124(5):1496–1548. doi: 10.1086/702007

[26] Manyika J, Lund S, Bughin J. Digital Globalization: The New Era Global Flows. McKinsey Global Institute; 2016.

[27] Kinnunen J. Gabriel Tarde as a founding father of innovation diffusion research. Acta Sociologica. 1996;39(4):431–442. doi: 10.1177/000169939603900404

[28] Barry A, Thrift N. Gabriel Tarde: imitation, invention and economy. Economy and Society. 2007;36(4):509–525. doi: 10.1080/03085140701589497

[29] Elkins Z, Simmons B. On waves, clusters, and diffusion: A conceptual framework. The Annals of the American Academy of Political and Social Science. 2005;598(1):33–51. doi: 10.1177/0002716204272516

[30] Golub B, Jackson MO. Naive learning in social networks and the wisdom of crowds. American Economic Journal: Microeconomics. 2010;2(1):112–149.

[31] Grabisch M, Mandel A, Rusinowska A. On the design of public debate in social networks. Operations Research. 2022.

[32] Trehub SE, Becker J, Morley I. Cross-cultural perspectives on music and musicality. Philosophical Transactions of the Royal Society B: Biological Sciences. 2015;370(1664):20140096. doi: 10.1098/rstb.2014.0096 25646519 PMC4321137

[33] Verboord M, Brandellero A. The Globalization of Popular Music, 1960-2010: a multilevel analysis of music flows. Communication Research. 2018;45(4):603–627. doi: 10.1177/0093650215623834

[34] Achterberg P, Heilbron J, Houtman D, Aupers S. A cultural globalization of popular music? American, Dutch, French, and German popular music charts (1965 to 2006). American Behavioral Scientist. 2011;55(5):589–608. doi: 10.1177/0002764211398081

[35] Ferreira F, Waldfogel J. Pop internationalism: Has half a century of world music trade displaced local culture? The Economic Journal. 2013;123(569):634–664. doi: 10.1111/ecoj.12003

[36] Bello P, Garcia D. Cultural Divergence in popular music: the increasing diversity of music consumption on Spotify across countries. Humanities and Social Sciences Communications. 2021;8(1):1–8. doi: 10.1057/s41599-021-00855-1

[37] Backstrom L, Huttenlocher D, Kleinberg J, Lan X. Group formation in large social networks: membership, growth, and evolution. In: Proceedings of the 12th ACM SIGKDD international conference on knowledge discovery and data mining; 2006;p.44–54.

[38] Boyd R, Richerson PJ. The origin and evolution of cultures. Oxford University Press; 2005.

[39] Park M, Park J, Baek YM, Macy M. Cultural values and cross-cultural video consumption on YouTube. PLoS ONE. 2017;12(5):e0177865. doi: 10.1371/journal.pone.0177865 28531228 PMC5439684

[40] Goel S, Anderson A, Hofman J, Watts DJ. The structural virality of online diffusion. Management Science. 2016;62(1):180–196. doi: 10.1287/mnsc.2015.2158

[41] Aslam S. YouTube by the Numbers: Stats, Demographics & Fun Facts; 2023. Available from: https://www.omnicoreagency.com/youtube-statistics/.

[42] Tinbergen J. Shaping the World Economy: Suggestions for an International Economic Policy. The Twentieth Century Fund, New York, 1962

[43] Gomez Rodriguez M, Leskovec J, Balduzzi D, Schölkopf B. Uncovering the structure and temporal dynamics of information propagation. Network Science. 2014;2(1):26–65. doi: 10.1017/nws.2014.3

[44] Garlaschelli D, Loffredo MI. Patterns of Link Reciprocity in Directed Networks. Phys Rev Lett. 2004;93:268701. doi: 10.1103/PhysRevLett.93.268701 15698035

[45] Fagiolo G, Reyes J, Schiavo S. The evolution of the world trade web: a weighted-network analysis. Journal of Evolutionary Economics. 2010;20:479–514. doi: 10.1007/s00191-009-0160-x

[46] Rosvall M, Bergstrom CT. Maps of random walks on complex networks reveal community structure. Proceedings of the National Academy of Sciences. 2008;105(4):1118–1123. doi: 10.1073/pnas.0706851105 18216267 PMC2234100

[47] Kleinberg JM. Authoritative sources in a hyperlinked environment. Journal of the ACM (JACM). 1999;46(5):604–632. doi: 10.1145/324133.324140

[48] Berry H, Guillén MF, Zhou N. An institutional approach to cross-national distance. Journal of International Business Studies. 2010;41(9):1460–1480. doi: 10.1057/jibs.2010.28

[49] Leyshon A, Webb P, French S, Thrift N, Crewe L. On the reproduction of the musical economy after the Internet. Media, Culture & Society. 2005;27(2):177–209. doi: 10.1177/0163443705050468

[50] Wagner T, Rose M, Baccarella C, Voigt KI. Streaming killed the download star! How the business model of streaming services revolutionizes music distribution. Journal of Organizational Advancement, Strategic and Institutional Studies. 2015;7(1).

[51] Wikström P. The music industry: Music in the cloud. John Wiley & Sons; 2020.

[52] Thompson B, Roberts SG, Lupyan G. Cultural influences on word meanings revealed through large-scale semantic alignment. Nature Human Behaviour. 2020;4(10):1029–1038. doi: 10.1038/s41562-020-0924-8 32778801

[53] Savage PE, Brown S, Sakai E, Currie TE. Statistical universals reveal the structures and functions of human music. Proceedings of the National Academy of Sciences. 2015;112(29):8987–8992. doi: 10.1073/pnas.1414495112 26124105 PMC4517223

[54] Park M, Thom J, Mennicken S, Cramer H, Macy M. Global music streaming data reveal diurnal and seasonal patterns of affective preference. Nature Human Behaviour. 2019;3(3):230–236. doi: 10.1038/s41562-018-0508-z 30953008

[55] World Bank. World Bank Open Data; 2023. https://data.worldbank.org/.

[56] Gaulier G, Zignago S. BACI: international trade database at the product-level (the 1994-2007 version). 2010. http://www.cepii.fr/CEPII/en/bdd_modele/bdd_modele_item.asp?id=37.

[57] United Nations. International Migrant Stock 2020: Destination and origin; 2020. https://www.un.org/development/desa/pd/content/international-migrant-stock.

[58] Vega SH, Mandel A. Technology diffusion and climate policy: a network approach and its application to wind energy. Ecological Economics. 2018;145:461–471. doi: 10.1016/j.ecolecon.2017.11.023

[59] Dueñas M, Fagiolo G. Modeling the international-trade network: a gravity approach. Journal of Economic Interaction and Coordination. 2013;8:155–178. doi: 10.1007/s11403-013-0108-y

