# Supplementary material for: The structure of global cultural networks: Evidence from the diffusion of music videos
Source: PLoS One. 2023 Nov 13;18(11):e0294149. doi: 10.1371/journal.pone.0294149 (PMC10642795; doi:10.1371/journal.pone.0294149)
Supplement: S1 Appendix — (PDF) [file pone.0294149.s001.pdf]

The structure of global cultural networks: evidence from the diffusion of music videos

Marco Dueñas[a,b,*] and Antoine Mandel[a]

[a]Centre d'Economie de la Sorbonne - Paris School of Economics- CNRS-Université Paris 1 Panthéon-Sorbonne, Paris, France
[b]AXES Research Unit, IMT School for Advanced Studies Lucca, Lucca, Italy
[*]Corresponding author email: marco.duenas@imtlucca.it

S1 Appendix

Supplementary descriptive statistics

The bulk of popular videos in a particular country can be used to evaluate the extent to which countries are open to more internationalized music. We define *international receptivity* as the proportion of videos in national charts that are also popular in other countries relative to the total number of videos available, i.e., the total variety. Although most countries have an international receptivity above 50%, indicating that the popular videos they consume are also popular in at least one other country, some countries have lower international content (Fig. S1A). In countries such as Turkey, Japan, Romania, Egypt, Israel, India, and Korea, only around 25% of popular videos are international. It is worth noting that most countries with low receptivity have unique languages–that are not spoken anywhere else. However, it can be said that cultural protectionism may also be a contributing factor (4). Therefore, while high receptivity may suggest that musical tastes are more internationalized, other factors, such as cultural features or policy interventions, may hinder the adoption of international content.

Additionally, there is considerable heterogeneity in how countries' levels of development relate to their international receptivity and total variety. Many middle-income countries have a high level of receptivity, whereas some high-income economies have lower receptivity. For instance, countries in Central America, such as El Salvador and Costa Rica, have a receptivity above 0.9, which is comparable to European countries like Luxembourg or Austria. Additionally, although most high-income economies listen to a vast collection of items, around 2,000, their total variety is comparable to lower-income countries in Africa (Fig. S1B).

Supplementary methods and results

Defining cascades

We use time series that contains the evolution of the number of weekly views of music videos in a given country. In order to model the information cascades, it is necessary to determine an

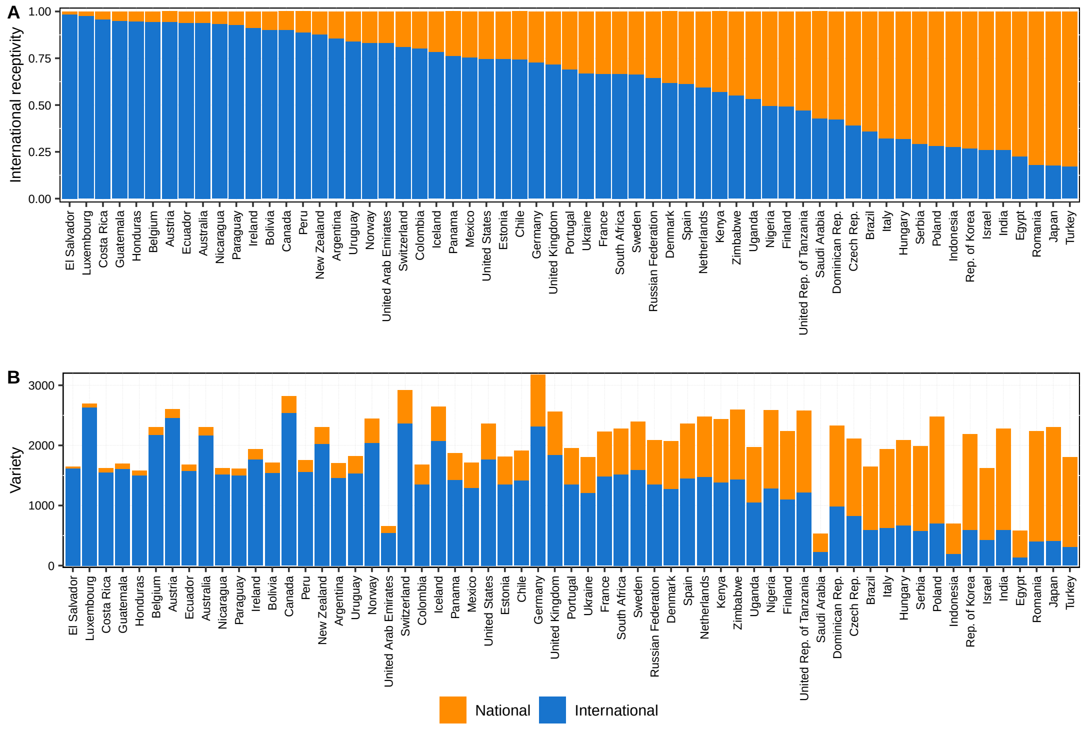

Fig. **S1**: **Country level descriptive statistics.** Panel A: Countries' receptivity. Panel B: Total variety of popular videos.

activation time, that is, the moment in which a given video is well-known in a country. We use the week when a video achieves the maximum number of views, which closely corresponds to the moment the adoption curve transits from early to late majority adopters. We tested another alternative, which was to use the first time a video appears in the country chart. The results are robust under both choices.

Network inference: the independent cascade model

Here, we comprehensively describe the network inference model based on Gomez-Rogriguez et al. (2). The method aims to determine the maximum likelihood network of influence between nodes based on the assumption that each cascade (i.e., the spread of information or behavior from one node to another) is an independent instance of a diffusion process. The diffusion process is modeled using a parameterized probability function that describes the likelihood of a node being activated by a parent node at a given time, assuming a certain transmission rate between them.

Each video c is characterized by a cascade of adoptions $\mathbf{t}^c = (t_1^c, ..., t_N^c)$, which is an N-dimensional vector of observed activation times. More precisely, for each node i, t_i^c is an element in $[t_0^c, t_0^c + T] \cup \{\infty\}$, which is equal to the time at which country i adopted the video c if finite and is infinite if the country did not adopt the video during a time interval of length T starting with the first adoption at time t_0^c. Note that a node assigned with ∞ as activation time does not mean that the node did not get activated but rather that its activation was not observed in the time window.

Let $\mathbf{C}$ be the set of cascades, one cascade for every music video, and denoted as $\mathbf{C} := \{\mathbf{t}^1, ..., \mathbf{t}^{|C|}\}$. We aim to infer from $\mathbf{C}$ an influence network consisting in a pair (G, A) where

$G = (V, E)$ is a graph (i.e., a set of nodes V and a set of edges E) representing the potential cultural influence paths and $A = [\alpha_{ji}]$ is a matrix of transmission rates, i.e., $\alpha_{ji} > 0$ quantifies how likely it is that a video spreads from node j to node i if $(j,i) \in E$ (and $\alpha_{ji} = 0$ if $(j,i) \notin E$).

Our approach assumes that each cascade in a network is an independent instance of a diffusion process. We use a parametric model to represent the diffusion probability from one node to another, parameterized by a transmission rate. To determine this transmission rate, we calculate the probability that another node activates a particular node at a given time based on the assumption that each node is either a parent or an offspring in the cascade. This probability is denoted as $f(t_i|t_j;\alpha_{ji})$, where α_{ji} represents the transmission rate between nodes j and i.

The functional form of $f(\cdot)$ makes structural assumptions about the diffusion process. In particular, we consider that once a video is popular in a country, the probabilistic rate at which this country influences another country is constant over time. This is equivalent to considering that the diffusion follows a Poisson process, leading to an exponential model for the conditional diffusion density over time. That is $f(t_i|t_j;\alpha_{ji}) = \alpha_{ji}e^{-\alpha_{ji}(t_i-t_j)}$, (if $t_j < t_i$ and zero otherwise). The corresponding log survival function is $\log S(t_i|t_j;\alpha_{ji}) = -\alpha_{ji}(t_i - t_j)$. As pointed out by Vega and Mandel (6), the Poisson assumption of a constant diffusion rate is a simple and natural benchmark in the absence of specific information about the dynamic aspects of the diffusion mechanisms in the fine-grained structure.

Therefore, with the conditional density $f(t_i|t_j;\alpha_{ji})$, we can determine the likelihood of a set of cascades $\mathbf{t}^1, ..., \mathbf{t}^{|C|}$ given a network $A = [\alpha_{ji}]$. This allows us to infer the maximum likelihood network. Thus, for a given cascade c, the likelihood of node i activation by note j is

$$f(t_i|t_1,...,t_N \setminus t_i; A) = \sum_{j:t_j<t_i} f(t_i|t_j;\alpha_{ji}) \times \prod_{j\neq k, t_k<t_i} S(t_i|t_k;\alpha_{ki}) \; ; \tag{1}$$

where $S(t_i|t_j;\alpha_{ji})$ is the survival (anti-cumulative distribution) function of edge $j \to i$, that is the probability that j does not cause i to activate by time t_i. Indeed, assuming a node gets activated only once, one shall consider it is activated by node j only if it has not been activated before by another node in the cascade.

One can then compute the likelihood of the activation in a cascade before time T as:

$$f(\mathbf{t}^c_{\leq T}; A) = \prod_{t_i \leq T} \sum_{j:t_j<t_i} f(t_i|t_j;\alpha_{ji}) \times \prod_{k:t_k<t_i, k\neq j} S(t_i|t_k;\alpha_{ki}) \; . \tag{2}$$

The likelihood of a cascade c, accounting for the fact that some nodes did not get activated, is

$$f(\mathbf{t}^c; A) = \prod_{t_i \leq T} \prod_{t_m > T} S(T|t_i;\alpha_{im}) \prod_{t_i \leq T} \sum_{j:t_j<t_i} f(t_i|t_j;\alpha_{ji}) \prod_{k:t_k<t_i, k\neq j} S(t_i|t_k;\alpha_{ki}) \; . \tag{3}$$

Finally, assuming that each cascade is independent, the likelihood of a set of cascades $\mathbf{C}$ is

$$f(\{\mathbf{t}^1, ..., \mathbf{t}^{|C|}\}; A) = \prod_{\mathbf{t}^c \in \mathbf{C}} f(\mathbf{t}^c; A) \; . \tag{4}$$

The objective of the network inference problem then is to find $A = [\alpha_{ji}]$, such that the likelihood of the observed set of cascades $\mathbf{C}$ is maximized. More precisely, we aim at solving the following

maximum likelihood optimization problem:

$$\min_{A} - \sum_{c \in \mathbf{C}} \log f(\mathbf{t}^c; A) \;; \qquad (5)$$
$$\text{subject to } \alpha_{ji} \geq 0, \text{for } i, j = 1, ..., N; i \neq j \;.$$

Considering the properties of the optimization problem in Eq. (5) it is possible to speed up a solution by using N distributed optimizations. For each node i, we can find the elements α_{ji} with $j = 1, ..., N \setminus i$. The local solutions lead to a globally optimal, as shown in (2), therefore each node's solution only requires the adoption times of other nodes in the cascades. Further details about the adopted estimations and the convex properties of the optimization problem can be found in (2).

Link-weight definition: after solving the network inference problem, the non-statistically significant parameters are set to zero under a significance level of 90%. There may be other theoretically zero parameters when there exists no possible path to connect two countries given the sample of cascades, for instance, in the case of two countries, i and j, where j does not appear in any cascade as an adopter before i, then $\alpha_{ji} = 0$.

Link weight, degree and strength distributions

We found that the link weights' distribution is highly skewed to the right (see Fig. **S2**A). This

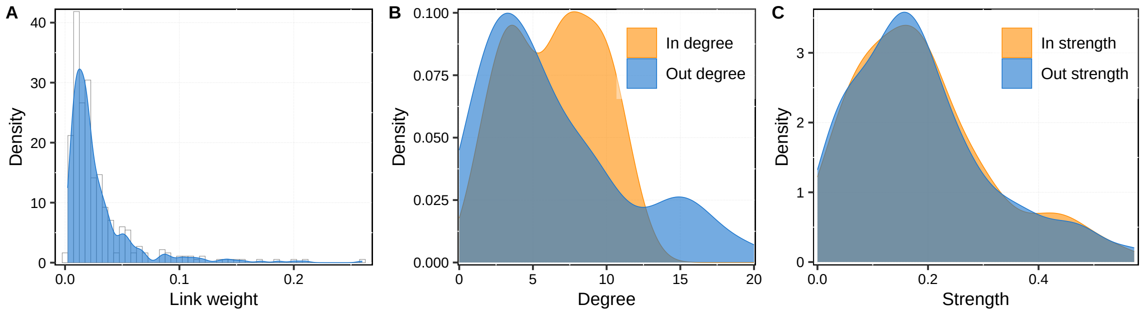

Fig. **S2**: **Network metrics: link weight, degree, and strength Distributions.** Panel A: Link weight density distribution. Panel B: In- and Out-degree density distributions. Panel C: In- and Out-strength density distributions.

suggests that many countries' relations are weakly connected while a few are relatively strongly connected. Additionally, in Figs. **S2**B and **S2**C, the probability distributions for node degree (in and out) and node strength (in and out) show that all these distributions are right-skewed, except the in-degree distribution. Overall, these findings indicate that in the network, typically, nodes have more incoming than outgoing neighbors, and a few countries are strong influencers or have been strongly influenced.

Fig. **S3** shows the scatter plots of the in- and out-node degree and node strength with the gross domestic product per capita (GDPpc). A positive and significant correlation between these statistics and GDPpc is present for in-degree and in-strength and not significant but positive for out-degree and out-strength and strength. This suggests that higher-income countries tend to have more incoming neighbors and more substantial information inflows. Considering that income correlates positively with other centrality measures implies that countries' economic

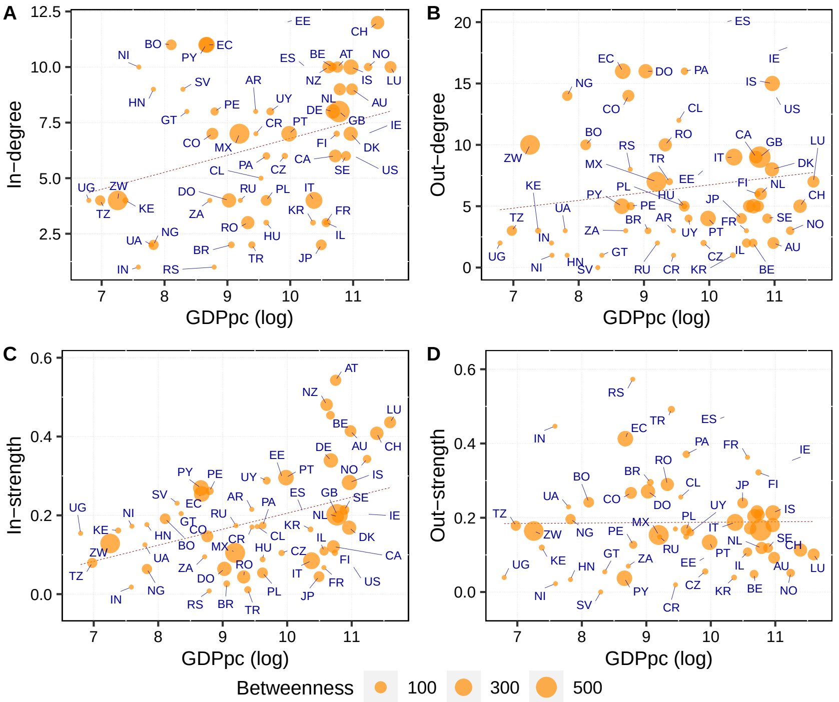

Fig. **S3**: **Node degree and strength correlations with GDPpc**. Panel A: In-degree. Panel B: Out-degree. Panel C: In-strength. Panel D: Out-strength. *Note*: Dot size is proportional to node's betweenness centrality.

status might impact the flow of information worldwide, but the relationship might be complex. Further evidence is presented with the results of the gravity model estimations below.

The cultural diffusion network (power-law diffusion)

We considered a power-law as an alternative functional assumption related to the diffusion process instead of the exponential form presented in the main text, it is $f(t_i|t_j;\alpha_{ji}) = \alpha_{ji}(t_i - t_j)^{1-\alpha_{ji}}$. This means that rather than assuming that the time to transmission is constant or follows an exponential distribution, the transmission occurs very quickly, but there is also a non-negligible chance of observing very long transmission times. The estimated network is presented in Figure S4.

The results are very similar to those from the exponential function presented in the methods section. Table S1 presents the estimated communities, demonstrating a very high similarity between the communities found with both assumptions.

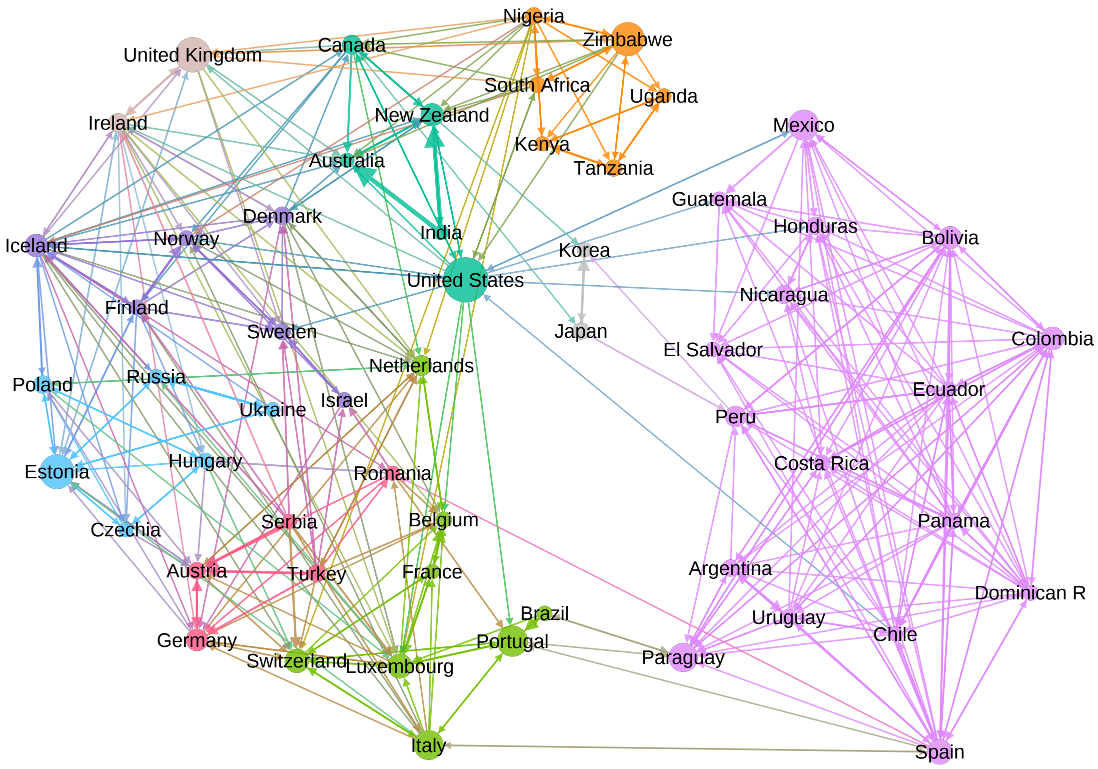

Fig. **S4**: **International cultural diffusion network (power-law diffusion assumption).** *Note:* nodes represent countries and links the most likely cultural diffusion channels, taking as a proxy the estimated transmission rates. Node's size indicates betweenness. Detected communities are depicted in different colors.

Table **S1**: Detected communities using the power-law diffusion assumption

Community members	Size
Argentina; Bolivia; Chile; Colombia; Costa Rica; Dominican Rep.; Ecuador; Spain; Guatemala; Honduras; Mexico; Nicaragua; Panama; Peru; Paraguay; El Salvador; Uruguay	17
Austria; Germany; Romania; Serbia; Turkey	5
Australia; Canada; India; New Zealand; United States	5
Belgium; Brazil; Switzerland; France; Italy; Luxembourg; Netherlands; Portugal	8
Czech Rep.; Estonia; Hungary; Poland; Russian Federation; Ukraine	6
Denmark; Finland; Israel; Iceland; Norway; Sweden;	6
United Kingdom; Ireland	2
Japan; Rep. of Korea	2
Kenya; Nigeria; United Rep. of Tanzania; Uganda; South Africa; Zimbabwe	6

Rapid diffusion videos

A relevant fact of the diffusion of music videos is that a considerable extent of music videos get popular very quickly worldwide. We identified 6,977 music videos with a presence in at least two countries' top 100 charts, and 4,490 of them were popularized in less than a week. The impossibility of having data in real-time, or at least with daily data (YouTube Charts are reported weekly), prevents the inference method from being applied considering those music videos. Here, we present an analysis to support the robustness of our results even when we cannot consider this part of the information.

We argue that even if we would have used more precise information, we would have found that the possibilities of influence between countries would continue to occur in similar neighborhoods of the already inferred network. That is, we want to show that these speedy videos are more likely to circulate between countries that are neighbors in the inferred network, and therefore there is little chance of these videos spilling over to different connections. Hence, there are no relevant reasons to think that countries' preferences depend on the videos' diffusion speed.

To achieve this, we analyze the similarity of the countries' speedy videos. Thus, let Ω be a set with the K music videos belonging to this diffusion domain. To compute the country-country similarity of musical tastes, we apply the Jaccard index (3), which is widely used as a relatedness measure to detect co-occurrences in data sets (5; 1). Therefore, for a country i, let y_i the vector in $\mathbb{R}^K$ where $y_{ik} = 1$ if video k belongs to the country top 100, and $y_{ik} = 0$ otherwise. The similarity matrix J between countries (i, j) reads

$$J_{ij} = \frac{\Lambda_{ij}}{\Lambda_i + \Lambda_j - \Lambda_{ij}}, \tag{6}$$

where $\Lambda_{ij} = \sum_{k\in\Omega} y_{ik}y_{jk}$ is the number of co-occurrences of songs in countries i and j, and $\Lambda_i = \sum_{k\in\Omega} y_{ik}$ is the total number of songs listed in country i. The resulting matrix J is used to define the country-country relatedness network, where nodes are countries and weighted links J_{ij} measure the similarity in music video tastes between them. Fig **S5** present the network representation of the Jaccard index matrix. A quick view allows us to observe similarities with the estimated diffusion network (Fig. 2 in the main text). To facilitate the comparisons, we have kept the same colors and communities of the inferred network.

Given that, by definition, any video that eventually diffuses from one country to another contributes to the formation of the similarity index, the similarity network will contain all the links that could be present in an inferred network A. So, our goal is to show that the similarity network restricted to the ties observed in the diffusion network, which we define as J^A, contains much of the information of the similarity network. Alternatively, the amount of similarities that spill over from the diffusion network is minimal. Thus, we calculate the participation of the node strength of the restricted similarity network to the inference network as follows

$$NS_i^A = \frac{\sum_j J_{ij}^A}{\sum_j J_{ij}} = \frac{\sum_j J_{ij} \cdot a_{ij}}{\sum_j J_{ij}} \tag{7}$$

where $a_{ij} = 1$ if there is influence in any direction between nodes i and j. We use the symmetric representation of the diffusion network since the Jaccard index is symmetric. Fig. **S6** shows that

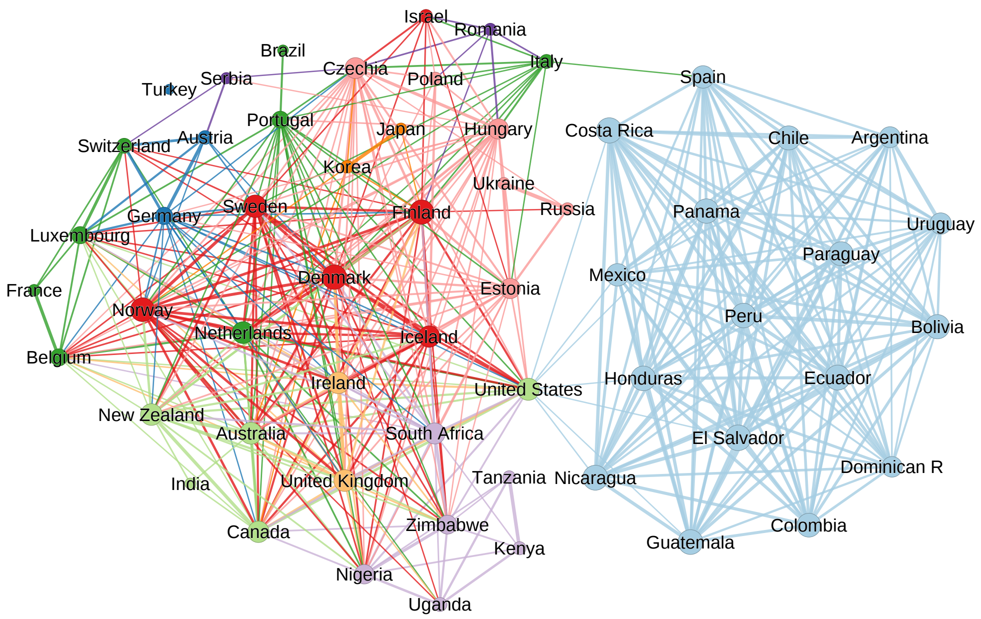

Fig. **S5**: **Similarity of countries' top music charts of speedy music videos.** *Note:* nodes represent countries and link the Jaccard similarity between countries' top music charts. In the figure, we omitted links with $J_{ij} < 0.15$ to enhance the visualization of the connections among the most similar countries.

node strength shares are high for most countries, which validates our arguments. However, in a few countries, such as India, Brazil, Israel, and Uganda, the shares are below 0.5.

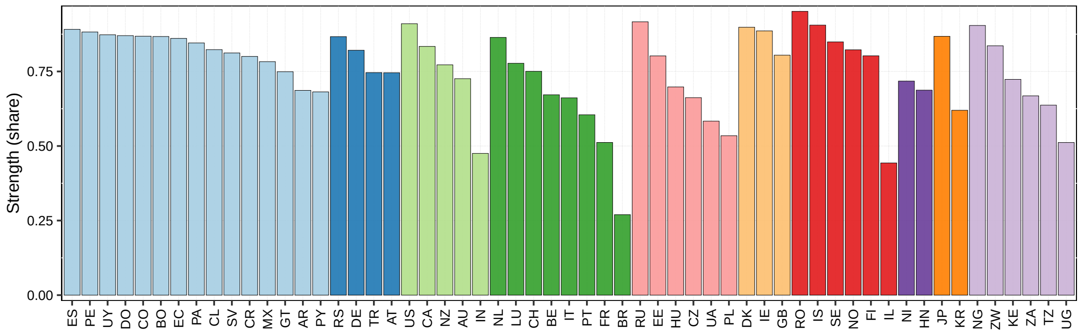

Fig. **S6**: **Link similarity projection on inferred links**

In conclusion, although with available information, we cannot determine the diffusion cascades for this group of videos, we suspect that they will most likely spread in the neighborhoods that we have already determined with the inference model.

Supplementary data information

Table **S2**: List of countries and ISO codes

ISO Code	Country	ISO Code	Country	ISO Code	Country
AR	Argentina	FR	France	NZ	New Zealand
AT	Austria	GB	United Kingdom	PA	Panama
AU	Australia	GT	Guatemala	PE	Peru
BE	Belgium	HN	Honduras	PL	Poland
BO	Bolivia	HU	Hungary	PT	Portugal
BR	Brazil	IE	Ireland	PY	Paraguay
CA	Canada	IL	Israel	RO	Romania
CH	Switzerland	IN	India	RS	Serbia
CL	Chile	IS	Iceland	RU	Russian Federation
CO	Colombia	IT	Italy	SE	Sweden
CR	Costa Rica	JP	Japan	SV	El Salvador
CZ	Czech Rep.	KE	Kenya	TR	Turkey
DE	Germany	KR	Rep. of Korea	TZ	United Rep. of Tanzania
DK	Denmark	LU	Luxembourg	UA	Ukraine
DO	Dominican Rep.	MX	Mexico	UG	Uganda
EC	Ecuador	NG	Nigeria	US	United States
EE	Estonia	NI	Nicaragua	UY	Uruguay
ES	Spain	NL	Netherlands	ZA	South Africa
FI	Finland	NO	Norway	ZW	Zimbabwe

References

[1] Campi, M., Dueñas, M., and Fagiolo, G. How do countries specialize in agricultural production? A complex network analysis of the global agricultural product space. *Environmental Research Letters 15*, 12 (2020), 124006.

[2] Gomez Rodriguez, M., Leskovec, J., Balduzzi, D., and Schölkopf, B. Uncovering the structure and temporal dynamics of information propagation. *Network Science 2*, 1 (2014), 26–65.

[3] Jaccard, P. Étude comparative de la distribution florale dans une portion des alpes et des jura. *Bulletin del la Société Vaudoise des Sciences Naturelles 37* (1901), 547–579.

[4] Kish, K. A. Protectionism to promote culture: South Korea and Japan, a case study. *U. Pa. J. Int'l Econ. L. 22* (2001), 153.

[5] Leydesdorff, L. On the normalization and visualization of author co-citation data: Salton's cosine versus the jaccard index. *Journal of the American Society for Information Science and Technology 59*, 1 (2008), 77–85.

[6] Vega, S. H., and Mandel, A. Technology diffusion and climate policy: a network approach and its application to wind energy. *Ecological Economics 145* (2018), 461–471.
